# The Improving Effects of Probiotic-Added Pollen Substitute Diets on the Gut Microbiota and Individual Health of Honey Bee (*Apis mellifera* L.)

**DOI:** 10.3390/microorganisms12081567

**Published:** 2024-07-31

**Authors:** Hyunjee Kim, Abdulkadir Yusif Maigoro, Jeong-Hyeon Lee, Olga Frunze, Hyung-Wook Kwon

**Affiliations:** 1 Convergence Research Center for Insect Vectors, 119 Academy-ro, Yeonsu-gu, Incheon 22012, Republic of Korea; beamed79@hanmail.net (H.K.); aymaigoro@gmail.com (A.Y.M.); jeonghyeon@inu.ac.kr (J.-H.L.); frunzeon@gmail.com (O.F.); 2 Department of Life Sciences, Incheon National University, 119 Academy-ro, Yeonsu-gu, Incheon 22012, Republic of Korea; 3 Division of Research and Development, Insensory Inc., 119 Academy-ro, Yeonsu-gu, Incheon 22012, Republic of Korea

**Keywords:** *Apis mellifera* L., probiotics, gut microbiome, honey bee health, pollen substitute diet

## Abstract

Honey bee (*Apis mellifera* L.) health is crucial for honey bee products and effective pollination, and it is closely associated with gut bacteria. Various factors such as reduced habitat, temperature, disease, and diet affect the health of honey bees by disturbing the homeostasis of the gut microbiota. In this study, high-throughput 16S rRNA gene sequencing was used to analyze the gut microbiota of honey bees subjected to seven diets over 5 days. *Lactobacillus* dominated the microbiota in all diets. Cage experiments (consumption, head protein content, and *vitellogenin* gene expression level) were conducted to verify the effect of the diet. Through a heatmap, the Diet2 (probiotic-supplemented) group was clustered together with the Beebread and honey group, showing high consumption (177.50 ± 26.16 mg/bee), moderately higher survival duration (29.00 ± 2.83 days), protein content in the head (312.62 ± 28.71 µg/mL), and diet digestibility (48.41 ± 1.90%). Additionally, we analyzed the correlation between gut microbiota and health-related indicators in honey bees fed each diet. Based on the overall results, we identified that probiotic-supplemented diets increased gut microbiota diversity and positively affected the overall health of individual honey bees.

## 1. Introduction

Honey bees (*Apis mellifera* L.), with their remarkable pollination abilities, are important contributors to promoting agricultural development and maintaining the ecological balance of nature. However, widespread honey bee colony losses are observed globally due to parasitic infections as well as various stressors [1]. Pathogen infections, pesticide exposure, and nutritional stress are major contributors [2]. Honey bees suffer from low nutrition and can transition to preforagers from nurse bees, reducing their survival [3].

Adequate colony nutrition, comprising sufficient protein and carbohydrate reserves, is critical for honey bees to withstand the challenges of modern apiculture [4]. Pollen, the primary source of protein and lipids, and nectar or honeydew, which provide carbohydrates, are essential components of honey bee nutrition [4]. The estimated daily pollen consumption of an individual adult honey bee ranges from 3.4 to 4.3 mg [5]. Consequently, the annual pollen requirement for an entire colony is estimated to be between 13.4 and 17.8 kg [5]. The term “beebread” refers to the pollen collected by honey bees, which is then processed, fermented, and stored in the hive to provide food for the nurse bees [6].

Beekeepers often employ artificial diets such as supplementary pollen (which includes pollen) or pollen substitutes (which excludes pollen) during periods of pollen scarcity to sustain colony strength [7]. However, we focused on developing a pollen substitute diet due to the expense of pollen and its potential to spread disease [8]. In early studies of developing pollen substitute diets, soy flour and brewer’s yeast were used as protein supplements due to their commonality, affordability, and accessibility as protein sources [8]. An effective pollen substitute diet should appeal to honey bees’ taste preferences while benefiting their overall health [9].

Dietary nutrition profoundly influences various aspects of honey bee biology, including lifespan, immunocompetence, resistance to pathogen infection, behavioral transitions, and the composition of their microbiome, which occupies a significant portion of the honey bee gut [10]. The importance of microbiomes in honey bee health has recently gained considerable attention [11]. Unlike humans, where gut bacteria are diverse, approximately 95% of the gut bacteria in honey bees constitute the core microbiome [12]. This core microbiome consists of five core bacterial species belonging to *Alphaproteobacteria*, *Betaproteobacteria*, *Gammaproteobacteria*, *Firmicutes*, and *Actinobacteria* [13].

The composition of the honey bee microbiota continually changes according to developmental stage, diet, and environment [14,15,16]. These changes influence the health status of honey bees by either strengthening or weakening their immunity, rendering them more or less susceptible to disease [17,18]. Pathogens and parasites are biological agents that can cause disease, significantly impacting the microbiome by affecting immunity, inducing oxidative stress, and altering behavior [14,15,16,17,18]. For instance, a high abundance of *γ-proteobacteria* was observed in the gut microbial community of honey bees undergoing colony collapse disorder (CCD) [19]. This elevated presence of *γ-proteobacteria* may have detrimental effects on honey bee health or play a beneficial role in enhancing the resilience of insect hosts against gastrointestinal threats [19].

Conversely, an increased abundance of *Lactobacillus* and Actinobacteria (*Bifidobacterium*) was observed in diseased honey bees [20]. These symbiotic microbes can adapt to various microenvironments and play a protective role against microbial invasion in honey bees [21,22]. This might suggest that an elevated amount of *Lactobacillus* and *Bifidobacterium* may be beneficial in improving host immunity to honey bee damage. Similarly, the abundance of these microbiomes correlates with the significant enrichment of carbohydrate metabolism in diseased honey bees [23].

Several delivery mechanisms have been developed for administering probiotics to bees through their diet, including the integration of probiotics into pollen patties, suspension in sucrose syrup, and the application of advanced spray-based formulations [24]. There are several studies about the mechanisms by which diet influences gut microbiota [25,26]. The results showed that probiotics including sugar syrup led to a higher abundance of beneficial bacteria in the gut of honey bees [25]. In another study, the result showed that different artificial diets affect gut microbiota abundance [26]. Diet has pronounced effects on shaping the gut microbiota in insect species [27,28].

*Vitellogenin* (*Vg*), a glycolipoprotein produced in fat body cells, serves pivotal functions in protein regulation and storage [29,30]. It is considered a biomarker for assessing dietary quality and nutritional status, influencing various aspects of honey bee biology including longevity, immune response, and caste differentiation [9,31,32]. The protein content in the head correlates with hypopharyngeal gland development, which is essential for producing protein-rich secretions like royal jelly, which is crucial for larval feeding and colony sustenance [33,34]. Although the response of honey bee gut microbiota to different diets is influenced by various factors such as season, temperature, developmental stage, and diseases, its role remains a subject of discussion. Diet significantly shapes the honey bee gut microbiota, impacting health stability and immune response against diseases and pathogens. In this study, we investigated the effects of pollen substitute diets on gut microbiota and evaluated individual honey bee performance, including survival, consumption, head protein content, *Vg* gene expression level, and diet digestibility.

## 2. Materials and Methods

### 2.1. Diet Preparation

In our previous study, we developed pollen substitute diets through field performance testing [9]. Subsequently, four diets were formulated: Diet1, Diet2, Diet3, and Diet4. The specific composition and concentrations of each diet are mentioned in detail Table 1. In our formulations, inosine-5’-monophosphate (IMP) and guanosine-5’-monophosphate (GMP) were included to enhance umami taste reactions, procured from Sigma Aldrich (Milwaukee, WI, USA) [35]. Apple juice and chlorella were incorporated as functional additives in some diet formulas. Apple juice (Jaan Company, Seoul, Republic of Korea) served as a longevity support ingredient, resulting in increased longevity among adult codling moths, while *Cydia pomonella* [36] Chlorella (Cheonil Herbal Medicine, Daegu, Republic of Korea) was included to promote colony formation and boost *Vg* synthesis in honey bees. Soytide and M were added as probiotic supplements to certain diet formulations [37]. Soytide (CJ Global Food and Bio Company, Seoul, Republic of Korea) is a fermented soybean flour rich in protein, with minimal anti-nutritional and allergenic properties, supporting both development and efficient nutrient utilization. In our previous study, we developed M, which is another fermented soybean flour utilizing the *Bacillus subtilis* strain that exhibits outstanding enzyme activity, particularly in cellulase and amylase [37]. Furthermore, it demonstrates potent antimicrobial properties against *Paenibacillus* larvae. Similar to other probiotics for honey bees, Soytide and M degrade allergenic proteins such as *β-conglycinin* and *glycinin*.

Megabee (Castle Dome Solutions, Helena, AR, USA) is a widely used commercial diet among beekeepers in the US. It comprises a blend of plant-based proteins without pollen and serves as a positive control [38]. To mimic natural colony conditions, beebread and honey diet forms were utilized by cutting from a frame of a colony, as they represent the natural diet of honey bees in their colony’s environment. Meanwhile, 60% syrup (Control) was employed as a negative control [39].

### 2.2. Cage Experiment and Sampling

**Figure 1:**
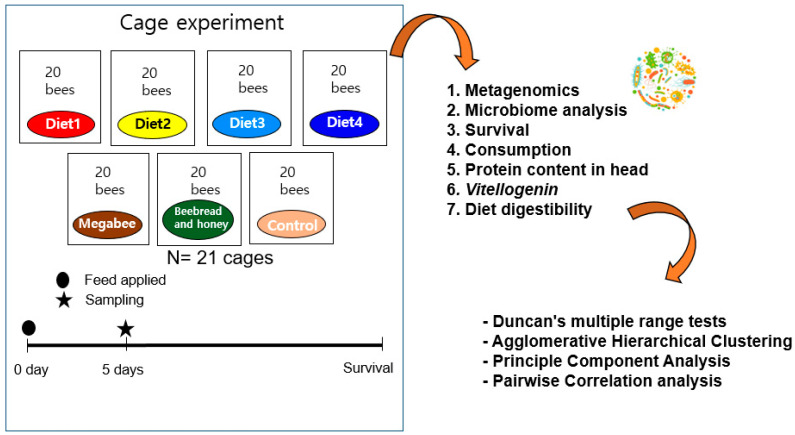
Schematic overview of experimental design. For this experiment, 21 cages were utilized, with 20 honey bees in each cage. The diets were provided to the honey bees for their entire lifespan. However, for the molecular experiment, honey bees were sampled after being fed each diet for 5 days.

The cage experiment was conducted for five days at 33 ± 2 °C and 55 ± 5% relative humidity conditions in an incubator. We chose three different healthy colonies from the Incheon National University Apiary; then, we took three capped brood frames from three colonies and carried them to an incubator. Each replication was conducted using each colony source. One cage (17 × 14.5 × 9.5 cm) was utilized for every twenty honey bees that newly emerged within 24 h from the carried frames (Figure 1). Three independent cages were used for each diet, and 20 g of each diet was provided with boiled water freely dispensed through a hole pierced in the cap of a 15 mL tube using a pin. The tube was then placed upside down on the top of the cage.

Survival was checked daily, and honey bees were removed from the cage accordingly. The quantity of diet consumption was measured by calculating the difference between 24 h and dividing it by the number of live honey bees for that day. The daily consumption was then summed over the five days of the experiment. 

Samples were collected at 5 days post diet introduction. We sampled 10 live honey bees from each cage to keep in a 15 mL tube and a total of 30 honey bees were sampled from each diet group. The samples were stored in a liquid nitrogen container and kept at −80 °C until the experiment. To evaluate digestibility, water-soluble protein concentrations in the hindgut contents of 9 sampled honey bees (3 honey bees × 3 cages) were compared to those in each respective diet [40]. Their head and hindgut were extracted and weighed. The diets were weighed at 200 mg for water-soluble protein concentration measurements. Following sample preparation with 0.25 M Tris-HCl buffer at pH 7.5, homogenization was performed using a sterile disposable homogenizer included in the Ultra Grinder B kit (Taeshin Bioscience Co., Ltd., Busan, Republic of Korea), along with 100 µL of buffer. The resulting mixture was then diluted to a 20% solution with the same buffer and centrifuged at 13,000 rpm for 30 s. The supernatant obtained was utilized for water-soluble protein concentration measurement. The water-soluble protein content was quantitatively assessed using the Pierce Bicinchoninic Acid (BCA) Protein Assay Kit (Thermo Fisher Scientific Inc., Waltham, MA, USA) [41], with absorbance measured at 562 nm using a SynergyTM HTX Multi-Mode Reader (Agilent, Santa Clara, CA, USA) [42]. The water-soluble protein content in the tissues was expressed as µg/mL. The approximate digestibility was then calculated using the formula (A − B)/A × 100, where A is the total water-soluble protein concentration in the diet and B is the total water-soluble protein concentration in the hindgut [40,43].

### 2.3. DNA Extraction

Nine honey bees were used for microbiome analysis based on each diet group. Before dissection, the honey bees underwent a 1 min surface sterilization in 70% ethanol, followed by dissection in PBS. The whole gut of each of the three honey bees was used to extract genomic DNA from their respective tube. Under aseptic conditions, whole-genome DNA was extracted using the Power soil kit (47014; QIAGEN, Hilden, Germany), according to the manufacturer’s instructions.

### 2.4. RNA Extraction and Quantitative PCR (qPCR)

Nine honey bees were obtained from each diet sampling tube, and the abdomens were used to extract RNA using a Qiagen RNeasy Mini Kit (#74104; Qiagen, Valencia, CA, USA). We analyzed *Vg* gene expression levels using quantitative PCR (qPCR) with cDNA templates from total RNA. For cDNA synthesis, 1 μg of total RNA was combined with oligo-dT and Invitrogen Superscript III enzyme. qPCR was performed on the Applied Biosystems StepOne Plus system with SYBR green qRT-PCR Master Mix from Fermentas. The cycling conditions included initial denaturation at 95 °C for 5 min, followed by 40 cycles of denaturation at 95 °C for 30 s, annealing at 60 °C for 30 s, and extension at 72 °C for 30 s. Details of qPCR primers are provided in Table S1. The results were normalized to the Control gene *β*-Actin using the 2^−ΔΔCt^ method [35], and each biological replicate underwent technical triplicate testing.

### 2.5. Sample Preparation, 16s Sequencing, and Taxonomic Analysis

For all samples, the National Instrumentation Center for Environmental Management (NICEM, www.nicem.snu.ac.kr, accessed on 22 September 2023), Republic of Korea, performed commercial PCR amplification, sample processing, and 16S rRNA gene sequencing. The samples were amplified using the KAPA HiFi HotStart ReadyMix (kk2601; Roche, Basel, Switzerland) and primers for the V3−V4 region of the 16S rRNA gene (Table S2). The following were the PCR conditions: 3 min at 96 °C, then 30 cycles of 30 s at 96 °C, 30 s at 55 °C, 30 s at 72 °C, and finally 5 min at 72 °C. All the PCR results were then performed on 1.2% agarose gels to determine band size and intensity. Ampure XP beads (A63882; Beckman, CA, USA) were used to purify amplified DNA from each sample. According to the content of DNA and molecular weight, the samples were pooled in identical quantities and utilized to create Illumina DNA libraries. The libraries were then sequenced in Illumina MiSeq runs to obtain 2 × 300 bp paired-end reads. The SILVA database v138.1 was used for taxonomic analysis. Protocol was adopted according to [44].

### 2.6. Analysis in QIIME2

The sequencing data were analyzed using the Quantitative Insights into Microbial Ecology (QIIME2) pipeline [45]. The raw reads were denoised and trimmed using the DADA2 pipeline [46]. All of the data were individually denoised before being merged for further analysis. The QIIME2 diversity plugin was used to construct the alpha and beta diversity index. The sampling depth was set at 3800. The bacterial diversity of honey bees from different diet groups was compared using a Shannon index. Using QIIME2, the Shannon diversity index for honey bee samples was calculated using bacterial OTU count data. The Shannon diversity index calculates species abundance values in the measurement of biodiversity and evaluates species heterogeneity. The Kruskal–Wallis H test was used to compare these results among developmental stages. To discover significant differences in their bacterial profiles, pairwise PERMANOVA was used with the “beta-group-significance” tool in QIIME2.

### 2.7. Bacterial Profiles

The read count and abundance data for bacterial OTUs were evaluated at the class, family, and genus levels. Low abundance taxa with a value of less than one percent were categorized as “ETC” from the dataset. Then, the relative abundance (RA) for each bacterial OTU was calculated across all samples. The average and maximum abundance of bacterial genera in the dataset were used to identify abundant bacterial genera.

### 2.8. Statistical Analysis

The statistical analyses including the Wilcoxon rank-sum test, PERMANOVA (permutational multivariate analysis of variance), and Kruskal–Wallis H test were performed using R version 4.1.0 in RStudio version 1.4.1106 and QIIME2 [47]. We conducted data analysis using SPSS version 25 (IBM). We employed Duncan’s multiple range tests to identify significant differences among means at a significance level of *p* < 0.05. The results were presented as mean values accompanied by their standard deviation (SD). Graphs were created using GraphPad Prism software (version 7.03). Microsoft Excel and XLSTAT software (Addinsoft Pearson Edition 2014, Addinsoft, Paris, France) were used for Principle Component Analysis (PCA), Agglomerative Hierarchical Clustering (AHC), and pairwise correlation analysis (*p* < 0.05). The mean, standard deviation, and variance of cage experiment indicators were calculated using descriptive statistics and visualized using the heatmap module.

## 3. Results

### 3.1. Reads Profiling for Microbial Community

Next-generation sequencing (NGS) returned an average of 71,394 quality trimmed reads with (~4000 bp maximum sequence depth). Out of 21 samples, Diet1 had three samples with a total of 230,294 filtered reads. Diet2, with three samples, possessed a total number of 242,331 filtered reads. Diet3, with three samples, possessed a total number of 223,433 filtered reads. Diet4 also had three samples, with a total of 225,041 filtered reads. Also, the Megabee group had three samples with a total of 209,476 filtered reads. The Beebread and honey group, possessing three samples, had a total number of 221,018 filtered reads. The control group had three samples with a total of 218,807 filtered reads (Table S3).

### 3.2. Gut Microbiota Diversity and Richness between Different Diet Groups

#### 3.2.1. Alpha Diversity

**Figure 2:**
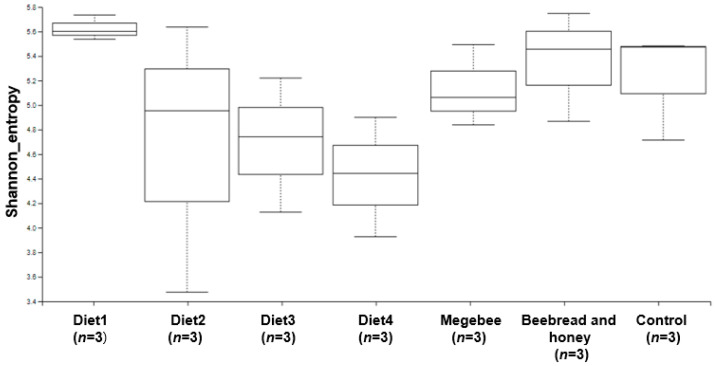
The Shannon entropy diversity index of the microbiome diversity based on each diet group sample. The box plot shows the diversity between and among the different diet groups.

To investigate whether different diet compositions can affect the microbial community in honey bees, the Shannon index was evaluated (Figure 2). The alpha diversity was calculated, which showed that there was a slight variation between Diet1 and Diet4 as well as between Diet3. Similar variation was observed between Diet4 and Control, as well as between the Beebread and honey group. The Shannon index was statistically analyzed among the diet combinations through the Kruskal–Wallis pairwise test (Table 2). The significant diet groups included Control compared to Diet2, Diet2 compared to Megabee, Diet2 compared to Diet3, and Diet2 compared to Diet1, with *p*-values of 0.049, respectively.

#### 3.2.2. Beta Diversity

**Figure 3:**
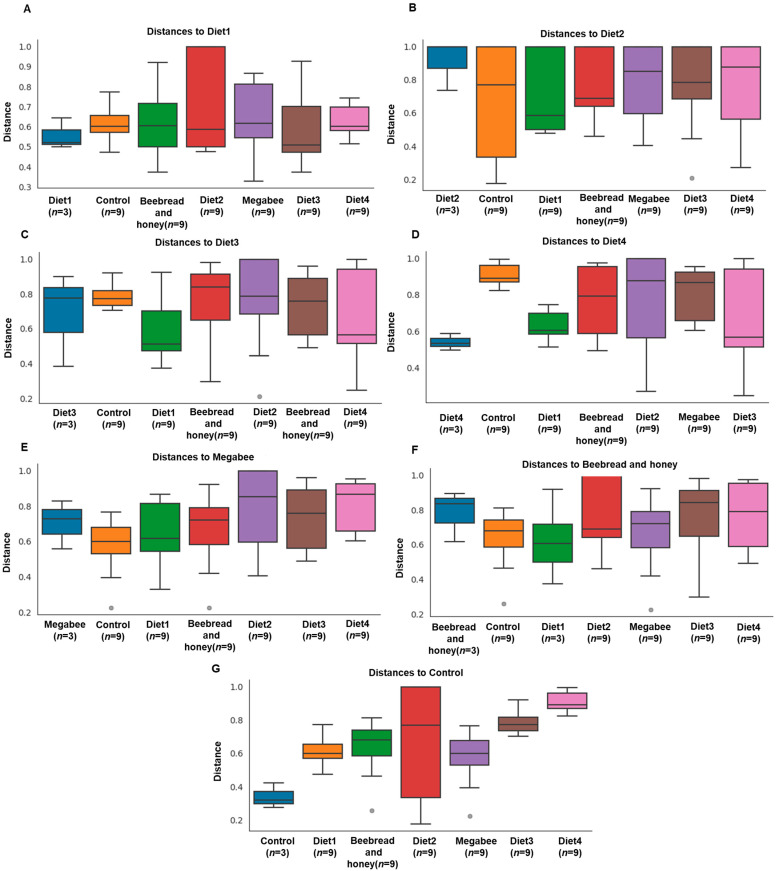
Beta diversity group significance of samples. Beta diversity of the microbiome in seven different diet groups including Diet1, Diet2, Diet3, Diet4, Megabee, Beebread and honey, and Control was analyzed. The beta diversity: unweighted UniFrac distance to Diet1 (**A**), Diet2 (**B**), Diet3 (**C**), Diet4 (**D**), Megabee (**E**), Beebread and honey (**F**), and Control (**G**) of the microbiome in the gut of *Apis mellifera* L. fed to these bees. N matches sample for each diet group. Diet1, Diet2, Diet3, Diet4, Megabee, Beebread and honey, and Control have 9 (=3 × 3), respectively.

Beta diversity metrics were calculated between the samples of varying diet groups to test the distance relationship between the diet groups. Boxplots of unweighted UniFrac were constructed, incorporating distances in phylogenic trees among the diet group members in comparison with other groups. The result was similar to the alpha diversity. We observed higher internal distances (mean: Diet1, 0.51; Diet2, 1.0; Diet3, 0.79; Diet4, 0.53; Megabee, 0.73; Beebread and honey, 0.82; and Control, 0.32) of the diet groups (Figure 3).

### 3.3. Gut Microbiota Taxonomy Diversity Based on Different Diets in Apis mellifera L.

**Figure 4:**
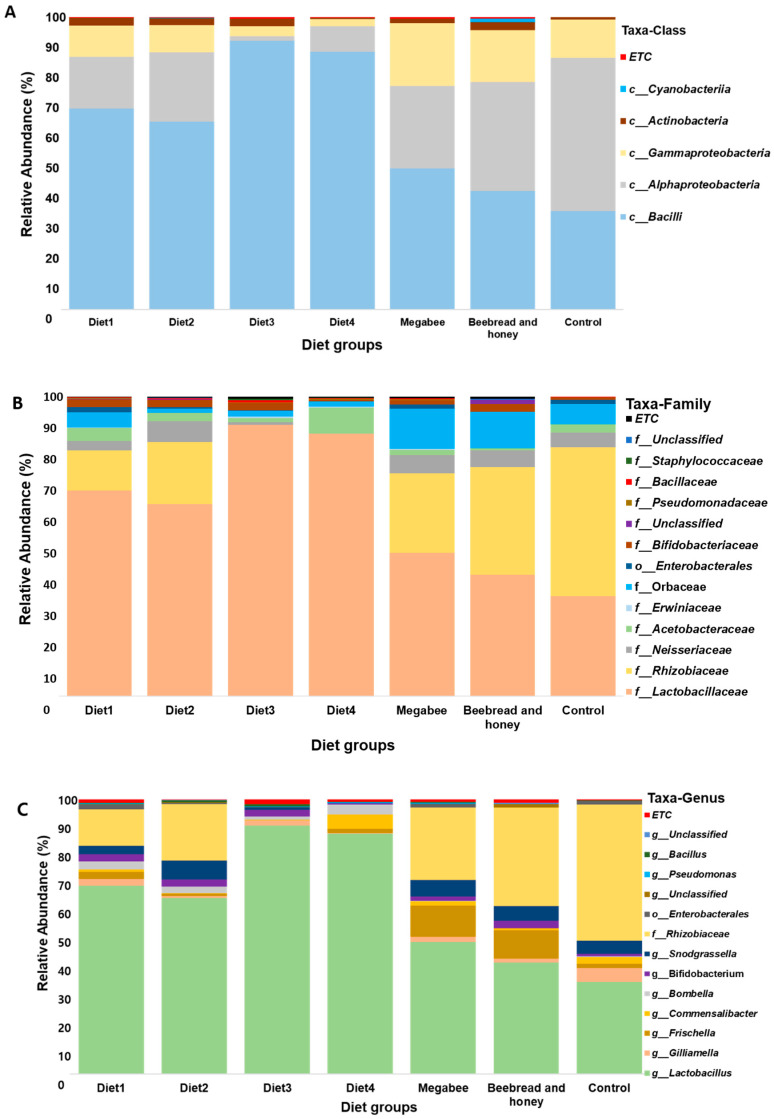
Microbiome profiles of *Apis mellifera* L. species using different diet compositions at class (**A**), family (**B**), and genus level (**C**), respectively. The bar height represents the average relative abundance of each taxon. The horizontal names represent the names of various diet groups. The microbiome composition was analyzed for each diet group including Diet1, Diet2, Diet3, Diet4, Megabee, Beebread and honey, and Control.

The microbiome profile was analyzed based on different diet groups, and at different taxonomic stages including class, family, and genus level, respectively (Figure 4A–C). At the class level, the most common bacteria, widespread among the different diet groups, were *Bacilli*, followed by *Alphaproteobacteria* and *Gammaproteobacteria* (Figure 4A). *Bacilli* was relatively more abundant in Diet3 (90% RA) and Diet4 (87% RA) compared to the other diets at the class level, including Control at around 33% RA. *Alphaproteobacteria* showed higher abundance in the Control group (>50% RA) and less abundance in the Diet3 (2% RA) and Diet4 (10% RA) groups. *Gammaproteobacteria* was relatively more abundant in the Megabee group. At the family level, the most common bacteria, widespread among the different diet groups, were *Lactobacillaceae*, followed by *Rhizobiaceae* (Figure 4B). Among the diet groups, *Lactobacillaceae* was relatively more abundant in the Diet3 (92% RA) and Diet4 (88% RA) groups. *Rhizobiaceae* showed higher abundance in the Control group (50% RA).

At the genus level, data were analyzed to identify the relevant bacterial operational taxonomic units (OTUs) distributed for each diet group. In total, 37 genera were found among all of the diet groups. The most abundant genera/taxa included *Lactobacillus* (62%), followed by *Rhizobiaceae* (21%), *Snodgrassella* (4%), and *Erwiniaceae* (4%). *Lactobacillus* not only occupied the highest abundance among the microbiome distribution, but also remained highly distributed among the diet groups and dominated the microbiome abundance in the Diet3, Diet4, Diet1, Diet2, and Megabee groups, respectively, but not in the Control or Beebread and honey groups (Figure 4C). *Rhizobiaceae* dominated the Control group, followed by Beebread and honey, Megabee, Diet2, and Diet1. *Frichella* was also widely distributed among all the diets, even though it was less abundant in most of the diets except the Megabee group and Beebread and honey group. Similarly, *Bifidobacterium* distribution was observed in all samples with almost similar abundance proportions.

**Figure 5:**
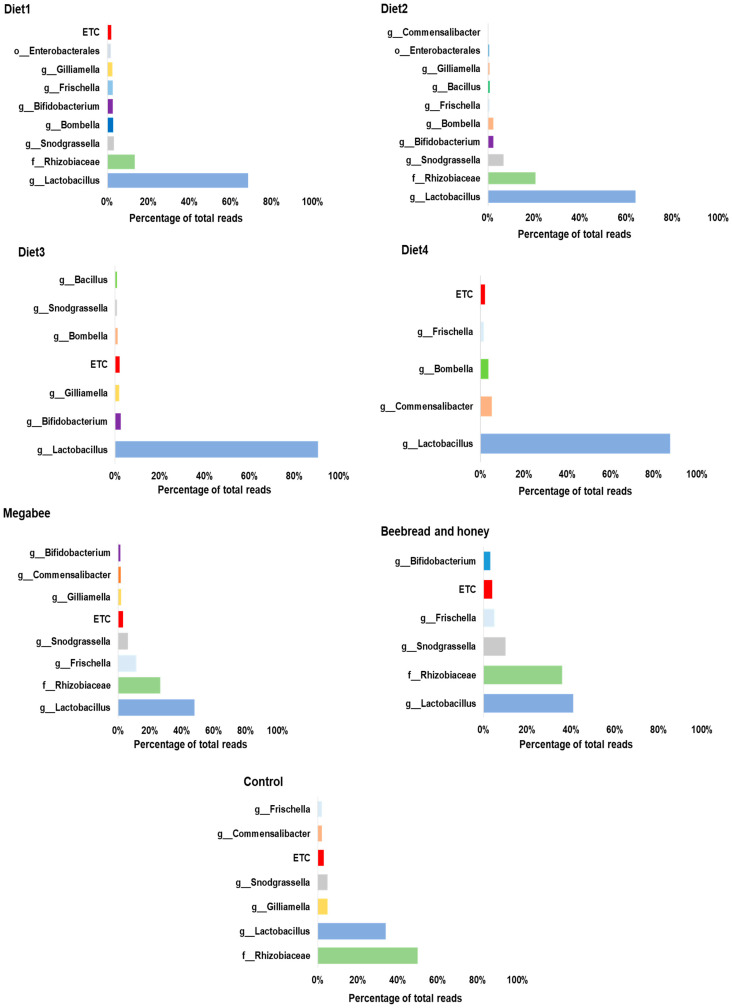
Microbiome profiles of *Apis mellifera* L. species using different diets at the genus level. The bar represents the percentage of total reads per diet group. The microbiome composition was analyzed for each diet group including Diet1, Diet2, Diet3, Diet4, Megabee, Beebread and honey, and Control. *Lactobacillus* shows the highest reads among the diet groups, except in the Control group with Rhizobiaceae with the highest reads.

Further, at the genus level, the percentage of total reads per diet was analyzed, and the microbiome distributions and proportions were filtered out according to the diet groups (Figure 5). Diet1 had *Lactobacillus* (69%) as the dominant genera, followed by *Rhizobiaceae* (13%). Diet2 group also showed *Lactobacillus* with the highest read (64%), followed by *Rhizobiaceae* (21%) and then *Snodgrassella* (7%). Diet3 showed the highest percentage read of *Lactobacillus* (91%) among the other diets, followed by *Bifidobacterium* (2%), and *Gilliamella* (2%). The Diet4 group showed the highest abundance of *Lactobacillus* (88%), followed by *Commensalibacter* (5%) and *Bombella* (4%). The Megabee group consisted of a large proportion of *Lactobacillus* (48%), followed by *Rhizobiaceae* (26%) and *Frischella* (11%). The Beebread and honey group showed an abundance of *Lactobacillus* (41%), followed by *Rhizobiaceae* (36%) and *Snodgrassella* (10%). For the Control, *Rhizobiaceae* was dominant at around (50%), followed by *Lactobacillus* with 34% and *Gilliamella* with 5%. Overall, among the 37 genera identified, five taxa, including *Lactobacillus*, *Commensalibacter*, *Frischella*, *Bifidobacterium*, and *Gilliamella*, were identified as being relatively high in abundance and widely distributed, with only one *Lactobacillus* identified in each diet group. Considering the overall distribution pattern, the abundance of *Lactobacillus* increased from 34% to 91% in Diet3; similarly, *Bifidobacterium* increased from (<2%) to 2% in Diet3. On the other hand, *Gilliamella* decreased from 5% to 2% in the same Diet3 group.

### 3.4. Cage Experiments Depend on Different Diets

**Figure 6:**
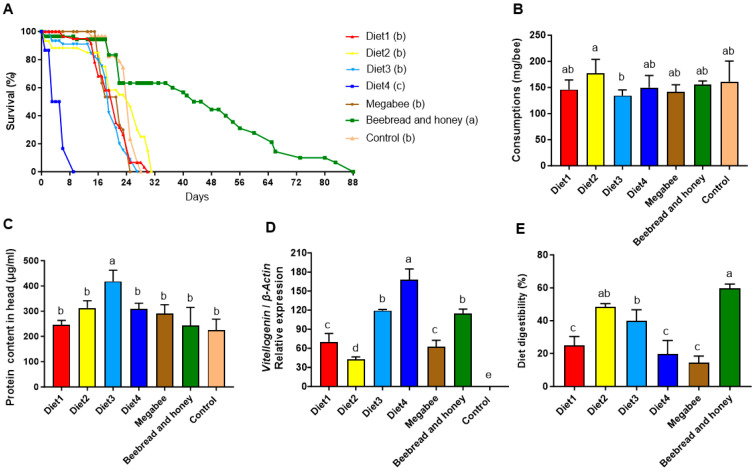
Bar plots showing the honey bee health effects based on the different diets from the cage experiment. (**A**) Survival. (**B**) Consumption. (**C**) Protein content in the head. (**D**) The *Vg* gene expression level. (**E**) Diet digestibility. Different letters (a, ab, b, c, d, e) after diet name in (**A**) and above bars indicate significant difference according to Duncan’s multiple-range tests (DMRTs) (*p* < 0.05) (mean ± SD, *n* = 3).

To evaluate the diet effect on honey bees, we estimated survival, consumption, protein content in the head, *Vg* gene expression level, and diet digestibility. Survival rates differed significantly between the diets (*p* < 0.05) (Figure 6A). The Beebread and honey group (85.50 ± 3.54 days) showed the longest survival, followed by Diet2 (29.00 ± 2.83 days), Diet1 (27.50 ± 3.54 days), Control (26.67 ± 1.53 days), Diet3 (24.33 ± 3.06), Megabee (24.00 ± 1.41 days), and Diet4 (7.50 ± 2.12 days). The cumulative consumption over five days also varied significantly between the diets (*p* < 0.05) (Figure 6B). Diet2 (177.50 ± 26.16 mg/bee) exhibited the largest consumption, followed by Control (161.00 ± 39.60 mg/bee), Beebread and honey (156.00 ± 6.56 mg/bee), Diet4 (149.50 ± 23.33 mg/bee), Diet1 (146.00 ± 18.38 mg/bee), Megabee (141.00 ± 14.14 mg/bee), and Diet3 (134.50 ± 10.61 mg/bee). After five days of feeding, the protein content in the head differed significantly between the diets (*p* < 0.05) (Figure 6C). Diet3 (418.44 ± 44.03 µg/mL) showed the highest protein content in the head, followed by Diet2 (312.62 ± 28.71 µg/mL), Diet4 (309.45 ± 22.10 µg/mL), Megabee (290.17 ± 35.38 µg/mL), Diet1 (246.63 ± 16.77 µg/mL), Beebread and honey (242.46 ± 72.62 µg/mL), and Control (223.75 ± 44.37 µg/mL). The *Vg* gene expression level also varied significantly between the diet groups (*p* < 0.05) (Figure 6D). Diet4 (167.57 ± 17.22) showed the highest *Vg* gene expression level, followed by Diet3 (119.44 ± 1.66), Beebread and honey (114.28 ± 7.28), Diet1 (70.16 ± 12.99), Megabee (62.44 ± 10.06), Diet2 (42.46 ± 3.95), and Control (1). The diet digestibility was significant between diet groups (*p* < 0.05) (Figure 6E). The Beebread and honey group (59.79 ± 2.44%) showed the highest diet digestibility, followed by the Diet2 group (48.41 ± 1.90%), Diet3 group (39.99 ± 6.57%), Diet1 group (25.02 ± 5.32%), Diet4 group (19.76 ± 8.18%), and Megabee group (14.49 ± 3.90%).

**Figure 7:**
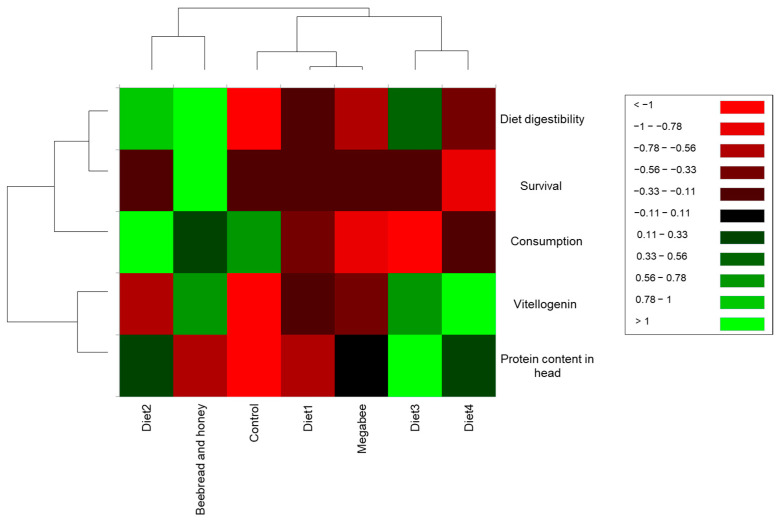
Heatmap with clustering depends on the several diets based on cage experiments.

We constructed a heatmap with clustering using experiment results depending on the different diet groups (Figure 7). The Diet2 group clustered with the Beebread and honey group, indicating that the experimental results for the Diet2 group were similar to those for the Beebread and honey group. Then, the Diet3 and Diet4 groups clustered together, but were separated from the other diets. This implies that the results of the experiments for Diet3 and Diet4 were similar to each other, but different from the rest of the diets. The Diet1 and Megabee groups clustered together, but were distinct from the Control group. Among the indicators, *Vg* gene expression level and protein content in the head clustered together, indicating a correlation or similarity between *Vg* gene expression level and protein content in the head. Diet digestibility and survival also clustered together, suggesting a relationship or similarity between those indicators. The clustering results provide insights into the relationships between different honey bee groups based on diets and experimental indicators.

### 3.5. Diet Group Distribution and Correlation

**Figure 8:**
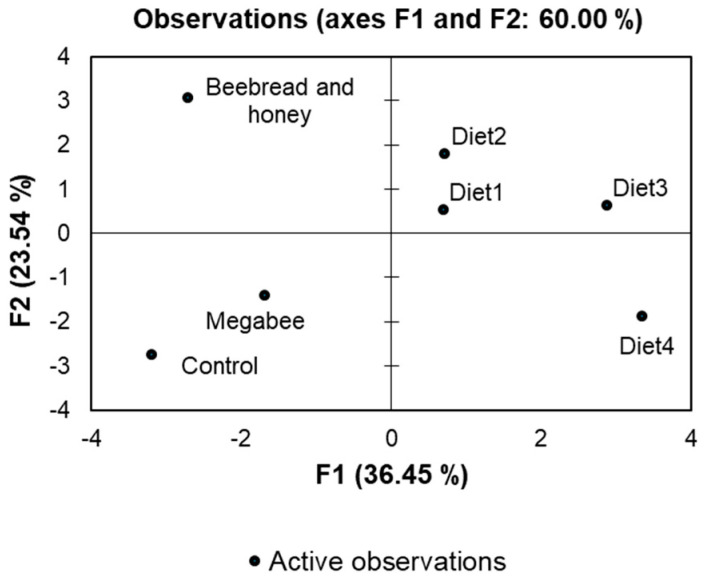
Distribution of honey bees’ microbiome and cage experiment based on various diet groups.

Each diet was distinguished through comparisons of gut microbiota and health performance using PCA (Figure 8). The discriminatory power of the F1 function was high (eigenvalue of 5.832), explaining 36.45% of the variation, with the second function (F2) contributing 23.54% of the variation. Among the indicators, g_*Lactobacillus*, f_*Rhizobiaceae*, g_*Snodgrassella*, g_*Bombella*, and protein content in the head showed the most discriminating parameters, implying their importance in distinguishing between different diets based on gut microbiota and health performance.

### 3.6. Diet Group Correlation

**Figure 9:**
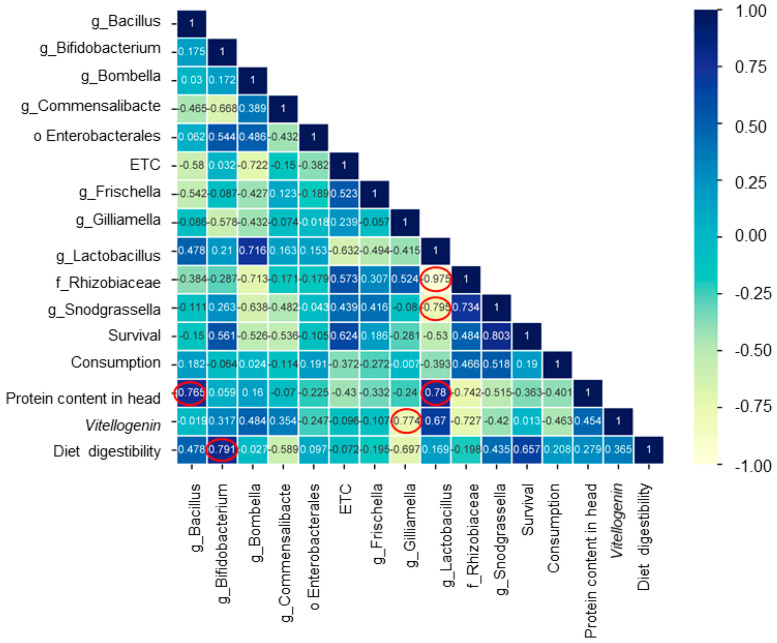
Heatmap of the correlation matrix using microbiome and cage experiment results depending on various diet groups. The red circle indicates statistical significance (*p* < 0.05).

A correlation matrix was constructed using microbiome profiles at the genus level and cage experiment results of pollen substitute diets (Figure 9). There were statistically significant correlations between some indicators (*p* < 0.05). Among them, g_*Lactobacillus* showed the most widespread correlations with three indicators including f_*Rhizobiaceae* (*r* = −0.975), g_*Snodgrassella* (*r* = −0.795), and protein content in the head (*r* = 0.78). g_*Bacillus* also exhibited a strong positive correlation (*r* = 0.765) with protein content in the head. Furthermore, a strong positive correlation (*r* = 0.791) was found between diet digestibility and g_*Bifidobacterium*. Additionally, a strong negative correlation (*r* = −0.774) was found between Vg gene expression level and g_*Gilliamella*.

## 4. Discussion

The significance of microbiomes goes beyond their interaction with the host; it includes their overall composition, metabolic products, and gene products, which are influenced by the host’s environment and lifestyle, including nutrition [48]. In honey bees, their core bacterial community dominates their gut as a whole [14]. Diet diversity increases the number of symbionts, which benefits the host [49]. Together, these influence the host’s biology, behavior, development, and immunity [50]. Different diet compositions could help us distinguish the best diet for health performance and a healthy microbiome community against opportunistic pathogens.

Diets with different compositions indirectly promote changes in the host-associated bacterial community [51]. Interestingly, our findings show that the beta diversity measurements of the relative abundance of bacteria between the diet groups have shown wide changes in microbiome compositions between the diet groups, specifically, Diet2.

In the past, a relationship between the content of the insect’s diet and bacterial diversity linked with the host was observed in different insect species, such as *Plodia interpunctella*, *Blattella germanica*, *Drosophila* spp., and *Lymantria dispar* [52,53]. From our study using *Apis mellifera* L., the bacterial communities associated with different diet compositions show fewer OTUs compared with the OTU abundance reported in other insects [51]. This may be a result of close compositions among the different diets used, except the Control, which has an entirely different composition with a higher sugar content and no protein.

The bacterial community within the honey bee gut is suggested to be malleable and influenced by various factors [14]. In this study, the diet groups showed a wide spectrum of bacterial distributions among the groups, including the Control group. The dominant microbiota present in the guts of honey bees were Proteobacteria, Firmicutes (*Bacilli*, *Lactobacillus*), Actinobacteria (*Bifidobacterium*), and Bacteroides. These taxa are consistent with those found in recent studies [54,55] suggesting that the dominant groups of bacteria in the honey bee guts may change little due to external factors such as temperature, seasons, toxins, and even nutrients such as sugar [56]. This is similar to our finding, whereby the dominant bacteria remained present, yet varied based on their relative abundance (RA) from the different diet types. For example, *Lactobacillus* was the most abundant genera in the Diet4 group, followed by *Commensalibacter* and *Bombella*. In contrast, Rhizobiaceae was the most abundant genera in the Control group, followed by *Lactobacillus* and *Gilliamella*. It is important to highlight the fact that researchers occasionally encounter unusual bacteria in the honey bee microbiome, such as the mentioned Rhizobium, which are well known as nitrogen-fixing bacteria found in the root nodules of legume plants [44]. These are likely *Gillamella* belonging to the order Rhizobium, yet they are considered part of the honey bee core microbiome [57]. This variation may be related to diet compositions, which can influence microbiome abundance. These changes in the microbiome favor the honey bee’s intestinal self-regulation system, allowing adaptation to environmental changes through varied dietary components.

At the genus level, not only did *Lactobacillus* (lactic-acid-producing bacteria, LAB) family members show higher abundance in the Diet3 group, but also in most of the other diet groups. The biological role of LAB, especially concerning insect physiology, is not fully elucidated. In the honey bee, LAB plays a protective role against pathogens [58], and supports respiratory requirements in cockroaches [59,60]. *Lactobacillus lactis* (LX3) enhanced the expression of genes responsible for antimicrobial peptides, improved productivity, and boosted the immune response in adult bees [61]. Therefore, a higher abundance of the LAB in different diet groups can be considered a marker for diet quality. From our findings, *Lactobacillus* bacteria dominate the Diet3 and Diet4 groups at the genus level with 91% and 87% of RA, respectively. While the Control group shows a low abundance of *Lactobacillus* with 34% of RA, the identification of *Lactobacillus* in the microbiota by diets is an interesting discovery because of its important role in insect biology [62]. Another study found a positive correlation between the presence of *Lactobacillus* and memory retention [63]. Compared to other bacterial genera, *Lactobacillus* likely boosts long-term memory due to its abundance of carbohydrate metabolism by phosphotransferase systems and production of glycerophospholipids, which are major components of neural membranes [64].

Recently, *Bifidobacterium* also became considered an LAB [65]. Both LAB and acetic acid bacteria (AAB) are common in the honey bee gut [66]. While the LAB increases honey bee immunity and protects the host from bacteria, yeast, and pathogens [67], host susceptibility to disease increases when the abundance of LAB and AAB is low [68]. Added to that, beekeepers use lactic and acetic acids to protect the honey bees against pathogens [69], indicating the vital role of these bacteria against the honey bee pathogens. This highlight the influence of some diet specific components in promoting microbiota changes since acid components are among the diets’ ingredients.

Among our developed diets, Diet2, Diet3, and Diet4 are probiotic-supplemented pollen substitute diets. The probiotics contribute to enhancing the gut health of honey bees by fostering the presence of beneficial bacteria. Soytide and M, specifically, are the major components that varied among the diet groups. Soytide, a soybean-derived peptide, has been shown to positively impact gut health in insects and overall well-being in honey bees [70]. On the other hand, unlike Diet2, Diet3, and Diet4, Diet1 does not contain the fermented Soytide component that is often used as a dietary supplement, and can significantly affect the microbiome of insects by introducing beneficial microorganisms [70,71]. This improvement in gut health is associated with other various benefits, including increased survival rate, colony strength, immunity, honey, and royal jelly production, as well as the development of the hypopharyngeal gland [24]. Furthermore, probiotics facilitate the efficient absorption of nutrients and the synthesis of essential compounds, thus supporting overall bee health. These endosymbionts play a crucial role in producing nutrients that may be lacking in their diet.

To validate the effects of probiotic-supplemented pollen substitute diets on honey bee health, we conducted cage experiments including survival rates, consumption patterns, protein content in the head, *Vg* gene expression levels, and diet digestibility. The Diet2 group was the only one of the pollen substitute diets we developed to exceed 30 days. The Beebread and honey group exhibited the longest survival of the diets tested at 88 days. This confirms that Beebread and honey is the best diet for honey bee survival in cage conditions. Previous studies have shown that honey bees can survive for about 30 days in cage experiments when protein supplementation is provided [72]. We confirm that the probiotics in Diet2 could make a positive contribution to honey bee survival. When examining the microbiota, the Beebread and honey group exhibited the highest *Snodgrasella* profile with 10%, followed by Diet2 with 7%. It is possible that the probiotics in Diet2 increased the *Snodgrasella* profile and subsequently improved survival rates. In contrast, the Megabee positive control group, despite having a *Snodgrasella* profile of 6%, showed a low survival rate, possibly due to low diet digestibility. When comparing the Megabee and Beebread and honey groups, both groups had similar consumption rates and protein content in the head. However, there were significant differences in *Vg* gene expression and diet digestibility, which ultimately led to a marked difference in survival rates in the cage experiment. The Diet2 group had the highest consumption, which is a key indicator of diet quality, attractiveness, and effectiveness [9]. This suggests that honey bees are likely to prefer this diet to others, including the Beebread and honey diet. However, consumption alone is not the only indicator of the overall value of a diet. While the amount consumed provides insight into the palatability and immediate acceptability of the diet, it does not necessarily reflect its nutritional adequacy or long-term benefits. Based on the survival and digestibility results observed in our study, it is clear that feeding honey bees the Beebread and honey diet, which closely mimics natural colony conditions, offers significant advantages over the formulated Diet2. Although Diet2 showed certain benefits, such as an improved *Snodgrasella* profile, the natural diet resulted in better overall survival rates. This indicates the superior nutritional quality and efficacy of the natural diet. While Diet2 is promising, particularly in terms of palatability, a good pollen substitute diet must not only be attractive to honey bees, but also have a positive impact on their health [8]. Future research should aim to further explore and refine diet formulations to optimize both palatability and comprehensive nutritional benefits, including their effects on the honey bee microbiota.

Diet3 showed the highest protein content in the head. Except for Diet3, the pollen substitute diets we developed had protein content in the head comparable to those in the Megabee and Beebread and honey groups. Protein content in the head is crucial for the development of the hypopharyngeal gland, which is situated in the head part, responsible for royal jelly secretion, and essential for larvae and queen nourishment [41]. Interestingly, despite Diet3 showing the lowest consumption level, it demonstrated moderately high levels of *Vg* gene expression. *Vg*, a yolk protein primarily synthesized in fat body, serves as the predominant nutrient storage molecule in honey bees. It is absorbed into the hypopharyngeal glands of nurse bees and converted into brood food [73]. *Vg* influences various aspects of honey bee behavior, physiology, and longevity [72,74], thus serving as a marker of honey bee health status. In the present study, our developed pollen substitute diets exhibited higher *Vg* gene expression levels compared to the Control group, which received a protein-free diet. This suggests that a protein source diet is essential for promoting *Vg* gene expression in honey bees. Our pollen substitute diets could potentially enhance *Vg* gene expression, thereby positively influencing hypopharyngeal gland development and honey bee health. However, Diet4 exhibited the highest *Vg* gene expression levels, but showed the shortest lifespan. Previous studies have suggested a positive correlation between *Vg* gene expression levels and honey bee lifespan [72,75]. This discrepancy might be attributed to the low level of diet digestibility within this group. Conversely, the Beebread and honey group, characterized by the highest diet digestibility, demonstrated the longest lifespan. Although the Megabee group showed higher *Vg* gene expression levels than the Diet2 group, it exhibited a lower survival rate. Further research is warranted to elucidate the relationship between diet digestibility and lifespan in honey bees.

When we constructed a heat map with clustering based on several diets using the cage experiment, we observed that the Diet2 group and the Beebread and honey group clustered together. This suggests that Diet2 closely resembles Beebread and honey, which are natural foods for honey bees. Beebread is the main source of protein, while honey is the main source of carbohydrates [4]. Furthermore, PCA analysis using all indicators, including microbiome and cage experiment results, showed that Diet2 closely clustered with the Beebread and honey group.

In the heatmap of the correlation matrix, *Bacillus* and *Lactobacillus* were positively associated with protein content in the head. The Diet3 group had the highest level of protein content in the head. It also had higher levels of *Lactobacillus* and *Bacillus* than the other diet groups. The dominance of *Bacillus* and *Lactobacillus* could potentially influence the absorption of protein content in the honey bee head through the gut–brain axis, thus contributing to honey bee health development [76]. *Bifidobacteria* showed a positive correlation with diet digestibility, and Diet2 exhibited high levels of diet digestibility and *Bifidobacteria*. The dominance of *Bifidobacteria* may enhance diet digestibility in the honey bee gut, potentially serving as a significant source of nutritional supply, thereby positively influencing honey bee health [77]. Notably, honey bee health is closely linked to the quality of consumed nutrition [77]. The Control group exhibited low protein content in the head and *Vg* gene expression levels, while the microbiota showed a high abundance of Rhizobiaceae. This finding is consistent with previous studies suggesting that honey bees exclusively fed sugar syrup tend to have elevated levels of Rhizobiaceae [78]. Additionally, overwintered honey bees exclusively fed sugar syrup also demonstrated a high level of Rhizobiaceae [79]. Rhizobiaceae have been associated with poor honey bee health [80].

Diet2 and Diet3 are characterized by a high abundance of *Lactobacillus*, *Bifidobacteria*, and *Snodgrasella*, which are recognized as key indicators of honey bee health [81]. However, in the cage experiment, Diet3 showed high levels of protein content in the head, *Vg* gene expression level, and diet digestibility, but survival rates were slightly low. On the other hand, the Diet2 group showed high levels of consumption and diet digestibility, but lower protein content in the head and *Vg* expression levels. Despite this, Diet2 showed a high survival rate, a critical health indicator. Our findings demonstrate that Diet2 is highly efficient in promoting individual honey bee health. Overall, our comprehensive study shows that probiotic-supplemented pollen substitute diets positively affect honey bee health. Notably, this research underscores the critical link between diet, microbiome, and honey bee health. Studies examining the impact of different diets on the gut microbiota have revealed significant alterations in its community structure. In honey bees, these changes influence their product quality, pollination activity, and health status, both positively and negatively. The application of next-generation technology enabled us to explore microbiome dynamics across various diet groups. Moreover, this observation remained consistent when assessing cage experiments. Collectively, this establishes a potential link between gut microbiota and the health status of individual honey bees. However, the study’s limitations include the relatively small sample size and the lack of field experiments. Conducting field experiments would provide more comprehensive insights, as honey bees would be exposed to their natural environment and foraging activities.

## 5. Conclusions

The present study demonstrated the impact of different diets on the microbiome community and its consequential effect on honey bee health. Comparative genomic analysis revealed that the probiotic-supplemented pollen substitute diet groups had higher abundances of *Bacillus*, *Lactobacillus*, and *Bifidobacterium*. These microbial indicators are associated with improved honey bee health when present in the appropriate proportions. The ability of LAB to increase honey bee immunity and protect the host from pathogens provides valuable insight into the identification of high-quality diets. The study shows a correlation between diet quality and individual honey bee performance, as indicated by the protein content in the head and diet digestibility. It also shows a positive correlation between the presence of *Bacillus*, *Lactobacillus*, and *Bifidobacterium*. Consequently, we propose that probiotic-supplemented pollen substitute diets, such as Diet2, are likely to provide better nutritional quality by shaping the microbiome community to a healthier state. Furthermore, verifying the effects of these diets at the colony level under field conditions is imperative. In addition, it is crucial to conduct colony-level experiments to investigate the relationship between diet, microbiota, and honey bee competency, as environmental factors can influence the abundance of microbiota.

## Figures and Tables

**Table 1:** The ingredients in pollen substitute diets remain consistent in their composition (%).

Ingredients	Diet1	Diet2	Diet3	Diet4
Brewer’s yeast	39.69	39.69	39.69	39.69
Egg yolk	2.21	2.21	2.21	2.21
Defatted soybean powder	2.21	-	-	-
Sugar	35.36	35.36	35.36	35.36
Boiled water	7.16	7.16	7.16	5.16
Canola oil	1.01	1.01	1.01	1.01
Cellulose	0.88	0.88	0.88	0.88
Wheat bran powder	0.88	0.88	0.88	0.88
Multiple vitamins	0.44	0.44	0.44	0.44
L-methionine	0.10	0.10	0.10	0.10
L-lysine	0.24	0.24	0.24	0.24
Citric acid	1.85	1.85	1.85	1.85
IMP	0.00	0.00	0.00	0.00
GMP	0.00	0.00	0.00	0.00
Tangerine juice	4.00	4.00	4.00	10.00
Soytide powder	-	-	2.21	2.21
Apple juice	4.00	4.00	4.00	-
Chlorella powder	-	0.08	0.08	-
M	-	2.21	-	-

IMP means inosine-5′-monophosphate, and GMP means guanosine-5′-monophosphate. M is a fermented form of defatted soybean flour instead of Soytide.

**Table 2:** Significant combination diet groups were detected using the Shannon index (Kruskal–Wallis pairwise test, *p* < 0.05).

Group 1	Group 2	*p*-Value
Control (*n* = 3)	Diet2 (*n* = 3)	0.049
Diet2 (*n* = 3)	Megabee (*n* = 3)	0.049
Diet2 (*n* = 3)	Diet3 (*n* = 3)	0.049
Diet2 (*n* = 3)	Dietl (*n* = 3)	0.049

## Data Availability

The raw sequence and metadata generated were submitted to the NCBI BioProject database under accession number PRJNA1074123.

## Supplementary Materials

The following supporting information can be downloaded at https://www.mdpi.com/article/10.3390/microorganisms12081567/s1: Table S1. Primer information for *Vg* and *ß-actin*; Table S2. Primer for amplifying V3–V4 region; Table S3. Table indicates the summary of the next-generation sequencing (NGS) analysis.
