# Supplementary material for: The Improving Effects of Probiotic-Added Pollen Substitute Diets on the Gut Microbiota and Individual Health of Honey Bee (Apis mellifera L.)
Source: Microorganisms. 2024 Jul 31;12(8):1567. doi: 10.3390/microorganisms12081567 (PMC11356693; doi:10.3390/microorganisms12081567)
Supplement: Supplementary file 1 [file microorganisms-12-01567-s001.zip › microorganisms-3123573-supplementary.pdf]

Table S1. Primer information for *Vg* and *ß-actin*

Gene		Primer sequence (5’-3’)	Size (bp)
Vg	F	GTTGGAGAGCAACATGCAGA	150
	R	TCGATCCATTCCTTGATGGT	
ß-actin	F	AGGAATGGAAGCTTGCGGTA	181
	R	AATTTTCATGGTGGATGGTGC	

Table S2. Primer for amplifying V3-V4 region

Primer	Sequence (5-------->3)
16S v34_F	TCGTCGGCAGCGTCAGATGTGTATAAGAGACAGCCTACGGGNGGCWGCAG
	TCGTCGGCAGCGTCAGATGTGTATAAGAGACAGNCCTACGGGNGGCWGCAG
	TCGTCGGCAGCGTCAGATGTGTATAAGAGACAGNNCCTACGGGNGGCWGCAG
	TCGTCGGCAGCGTCAGATGTGTATAAGAGACAGNNNCCTACGGGNGGCWGCAG
16S v34_R	GTCTCGTGGGCTCGGAGATGTGTATAAGAGACAGGACTACHVGGGTATCTAATCC
	GTCTCGTGGGCTCGGAGATGTGTATAAGAGACAGNGACTACHVGGGTATCTAATCC
	GTCTCGTGGGCTCGGAGATGTGTATAAGAGACAGNNGACTACHVGGGTATCTAATCC
	GTCTCGTGGGCTCGGAGATGTGTATAAGAGACAGNNNGACTACHVGGGTATCTAATCC

Sample-id	Input	Filtered	Percentage of input passed filter	Denoised	Merged	Percentage of input merged	Non-chimeric	Percentage of input non-chimeric
Diet1-1	112324	83303	74.16	82052	75119	66.88	7440	6.62
Diet1-2	98331	72881	74.12	71845	66993	68.13	10673	10.85
Diet1-3	101115	74110	73.29	72831	67794	67.05	8243	8.15
Diet2-1	121929	89586	73.47	88326	82205	67.42	11723	9.61
Diet2-2	101934	74692	73.27	74623	71058	69.71	11496	11.28
Diet2-3	104770	78053	74.5	76799	70603	67.39	11693	11.16
Diet3-1	103635	78153	75.41	77461	74256	71.65	9460	9.13
Diet3-2	96173	72330	75.21	71724	67975	70.68	12876	13.39
Diet3-3	100074	72950	72.9	72617	63419	63.37	9114	9.11
Diet4-1	82348	61006	74.08	60778	59577	72.35	14016	17.02
Diet4-2	116515	85883	73.71	85528	79363	68.11	10469	8.99
Diet4-3	110698	78152	70.6	77406	73649	66.53	8006	7.23
Megabee1	115335	85346	74	84205	77330	67.05	7094	6.15
Megabee2	87130	65763	75.48	64984	62078	71.25	7604	8.73
Megabee3	78792	58367	74.08	57877	55729	70.73	9310	11.82
Beebread and honey1	92773	71110	76.65	70123	66728	71.93	12586	13.57
Beebread and honey2	97375	72476	74.43	71428	65950	67.73	6860	7.04
Beebread and honey3	107285	77432	72.17	76315	71344	66.5	8047	7.5
Control1	116773	82815	70.92	81626	77141	66.06	11476	9.83
Control2	96009	69697	72.59	68837	64983	67.68	10024	10.44
Control3	91524	66295	72.43	65650	63733	69.64	12559	13.72

Table S3. The table indicates the summary of the Next-generation sequencing (NGS) analysis with a total of 21 samples. Diet1 samples (*n* = 3); Diet2 samples (*n* = 3); Diet3 samples (*n* = 3); Diet4 samples (*n* = 3); Megabee samples (*n* = 3); Beebread and honey samples (*n* = 3); and Control samples (*n* = 3). DADA2 output after removing the chimeric reads returned an average of 71,394 reads from the 21 samples.
